# Factors influencing advanced colorectal neoplasm anatomic site distribution in China: An epidemiological study based on colorectal cancer screening data

**DOI:** 10.1002/cam4.6722

**Published:** 2023-11-16

**Authors:** Kailong Zhao, Hongzhou Li, Baofeng Zhang, Wenwen Pang, Suying Yan, Xinzhu Zhao, Xinyu Liu, Wanting Wang, Qiurong Han, Yao Yao, Tianhao Chu, Zhiqiang Feng, Qinghuai Zhang, Chunze Zhang

**Affiliations:** ^1^ Department of Colorectal Surgery Tianjin Union Medical Center Tianjin China; ^2^ School of Medicine Nankai University Tianjin China; ^3^ Department of Gastroenterology Tianjin Union Medical Center Tianjin China; ^4^ Department of clinical laboratory Tianjin Union Medical Center Tianjin China; ^5^ School of Integrative Medicine Tianjin University of Traditional Chinese Medicine Tianjin China; ^6^ Tianjin Medical University Tianjin China; ^7^ The Institute of Translational Medicine Tianjin Union Medical Center Tianjin China; ^8^ Tianjin Institute of Coloproctology Tianjin China

**Keywords:** advanced colorectal neoplasms (ANs), anatomic site, colorectal cancer (CRC)

## Abstract

**Objective:**

Existing studies indicate that advanced colorectal neoplasms exhibit distinct clinical and biological traits based on anatomical sites. However, in China, especially for advanced colorectal neoplasms, there's limited information available on these traits. Our primary objective is to comprehensively study the characteristics of advanced colorectal neoplasm patients in different anatomical sites in China.

**Methods:**

We selected information from the colorectal cancer screening database in Tianjin, China, since 2010 as the study subject. We chose valid information from 3113 patients with comprehensive data and diagnosed advanced colorectal neoplasms (ANs) from a pool of 19,308 individuals to be included in the study. We then conducted further analysis to examine the correlation between these epidemiological data and tumor location.

**Results:**

Among the 3113 patients, neoplasms in the left side of the colon accounted for the largest proportion, while neoplasms in the right side of the colon had the smallest proportion, followed by rectal neoplasms. The highest proportion of advanced colorectal neoplasms was found among men. In the age group of 39–49 years old, the proportion of left late‐stage advanced colon neoplasms was equal to that of right late‐stage advanced colon neoplasms, while late‐stage advanced rectal neoplasms increased with age. Smoking, drinking, and a history of colon cancer in first‐degree relatives showed statistically significant associations with the location distribution of advanced colorectal neoplasms. A history of appendicitis, appendectomy, cholecystitis, or cholecystectomy did not significantly affect the location distribution of advanced colorectal neoplasms. However, among patients with such histories, there was a statistically significant relationship between advanced colon neoplasms on the right and those on the left and in the rectum. Similar results were observed for BMI.

**Conclusion:**

Our research findings demonstrate that advanced colorectal neoplasms display unique epidemiological characteristics depending on their anatomical locations, and these distinctions deviate from those observed in Western populations. These insights contribute to a more comprehensive understanding of the topic and offer valuable guidance for future research in China. We advocate for further investigations centered on the anatomical location of colorectal neoplasms to enhance the precision of colorectal cancer (CRC) screening and treatment.

## 1 INTRODUCTION

Colorectal cancer (CRC) is the second most prevalent cancer worldwide and the third most common disease overall.^1^ Its incidence is increasing, particularly among younger people; the incidence among those under 50 years old is on the rise.^2^, ^3^ Colorectal cancer has imposed a significant medical and health burden on both the world and China.^4^ Risk factors for colorectal cancer include both modifiable elements such as lifestyle, smoking, alcohol intake, and obesity, as well as non‐modifiable factors such as age and gender.^5^

The location of colorectal cancer can be divided into left‐sided colon cancer, right‐sided colon cancer, and rectal cancer. It has been observed that colorectal cancer (CRC) at different locations exhibits distinct biological properties and prognoses, which may be attributed to the varying embryonic origins of each site.^6^ Furthermore, gene mutations such as KRAS, PIK3CA, and BRAF mutations have a higher incidence rate in right‐sided colon cancer,^7^ while TP53 mutations are more commonly found in left‐sided colon and rectal cancers.^8^, ^9^ Studies have indicated that right‐sided colon cancer generally carries a less favorable clinical prognosis compared to left‐sided colon cancer.^10^, ^11^

Accessing characteristics and epidemiological data related to advanced colorectal neoplasms from different anatomical locations can enable local researchers to develop a comprehensive understanding of regional risk factors within the local population. This, in turn, can facilitate the implementation of more effective measures for disease prevention and treatment.^12^ While substantial progress has been made in developed countries,^13^, ^14^ research on advanced colorectal neoplasms in China, as a developing nation, remains limited. There is a scarcity of studies focusing on advanced colorectal neoplasms in different anatomical sites. Therefore, the primary objective of this study is to consolidate epidemiological insights into advanced colorectal neoplasms in China, categorized by anatomical location. This research aims to bridge this gap in knowledge and provide pertinent recommendations for the early prevention and treatment of individuals with advanced colorectal neoplasms in China. Thus, in this analysis, our focus was on the epidemiological characteristics of advanced colorectal neoplasms (AN) at different anatomic sites, considering factors such as age and gender, as well as examining the associations between lifestyle and family history of CRC with AN at different locations. These findings could contribute to a more nuanced characterization of colorectal neoplasms and inform improved strategies for CRC screening programs and clinical treatment.

## 2 MATERIALS AND METHODS

### 2.1 Data source

Since 2012, a large‐scale colorectal cancer screening program has been implemented in Tianjin, primarily focusing on local residents aged 40–74 years old. Participants initially completed a high‐risk questionnaire and underwent fecal immunochemical testing (FIT). Individuals with a positive result from either of these methods were subsequently advised to undergo a colonoscopy examination. Data collection involved gathering participants' results, including colonoscopy findings, as well as general and clinical information. This encompassed demographic characteristics (age, gender, weight, and height), additional details (past medical history, personal history, and surgical history), and pathological diagnoses, among other key information. We included an analysis of 3113 patients who were diagnosed with advanced colorectal neoplasms and had participated in colonoscopy examinations at Tianjin Union Medical Center without any missing information. All individuals provided informed consent prior to enrollment, and all study and methodology adhered to the principles of the Helsinki Declaration. This study was approved by the ethics committee of the Tianjin Union Medical Center.

### 2.2 Measurement and definition

All colonoscopies were performed by a group of skilled endoscopists licensed by the medical facility's Endoscopy Committee. Gastrointestinal pathologists validated all abnormal findings following the latest clinical standards. Successful colonoscopies were completed, and full pathology results were provided for participants. We specifically selected individuals with advanced colorectal neoplasia, including CRC and advanced adenomas (polyps larger than 10 mm, those with villous components, or exhibiting high‐grade dysplasia).

Furthermore, we collected all relevant questionnaire data in its entirety. Based on age, patients were divided into four groups: 39–49, 50–59, 60–69, and 70 years. Smoking status was categorized into three groups: never smoking, current smoking, and former smoking. Other variables were binary. We classified BMI values as normal weight or obese/overweight using a cutoff value of 25 kg/m^2^.

### 2.3 Classification of anatomical parts

In this investigation, we categorized advanced colorectal carcinoma sites into three groups: the right colon group (comprising the cecum, ascending colon, hepatic flexure, and transverse colon), the left‐colon group (which includes the splenic flexure, descending colon, and sigmoid colon), and the rectal group.

### 2.4 Data analysis

By using R software, we input information related to diagnosis, medical history, and personal details. We then categorized the selected population to determine the quantity and percentage of advanced colorectal neoplasms in different anatomical locations. Univariate analysis (Pearson's chi‐square test) was employed to assess the relationship between various patient characteristics (gender, age, smoking, alcohol consumption, BMI, family history of CRC, previous appendicitis or appendectomy, gallbladder inflammation or cholecystectomy) and the primary site of advanced colorectal neoplasms (left colon, right colon, rectum). Further, *p*‐values were calculated to compare population proportions and associated risk factors among all anatomical sites and subgroups of anatomical sites. Additional univariate analyses (Pearson's chi‐square test) were conducted to explore the detailed distribution of advanced colorectal neoplasm locations within certain segments of the colon. The left side includes the descending colon and sigmoid colon, while the right side includes the ascending colon and transverse colon. A two‐tailed test with *p* < 0.05 is considered statistically significant.

## 3 RESULTS

### 3.1 General patient characteristics

The characteristics of the 3113 patients included in this study with no missing information are shown in Table 1. The majority of the patients are from Northern China, with a higher number of male patients than female patients, and the most common age group is 60–69 years old.

**TABLE 1:** General information about the enrolled patients.

Characteristic	Number (*N* = 3113)	Percentage (%)
Gender		
Female	1210	38.9
Male	1903	61.1
Age		
39–49	51	1.6
50–59	499	16.0
60–69	1600	51.4
≥70	963	30.9
Smoking status		
Never	2145	68.9
Former	185	5.9
Current	783	25.2
Drinking frequency		
Never	2200	70.7
Ever	913	29.7
History of appendicitis or appendectomy		
No	2943	94.5
Yes	170	5.5
FHCRC		
No	2881	92.5
Yes	232	7.5
History of cholecystitis or cholecystectomy		
No	2919	93.8
Yes	194	6.2
BMI		
<25	1645	0.53
≥25	1468	0.47

Abbreviations: AN, advanced neoplasm; BMI, body mass index; FHCRC, Family history of CRC in FDR.

### 3.2 Advanced colorectal neoplasms localization

**FIGURE 1:**
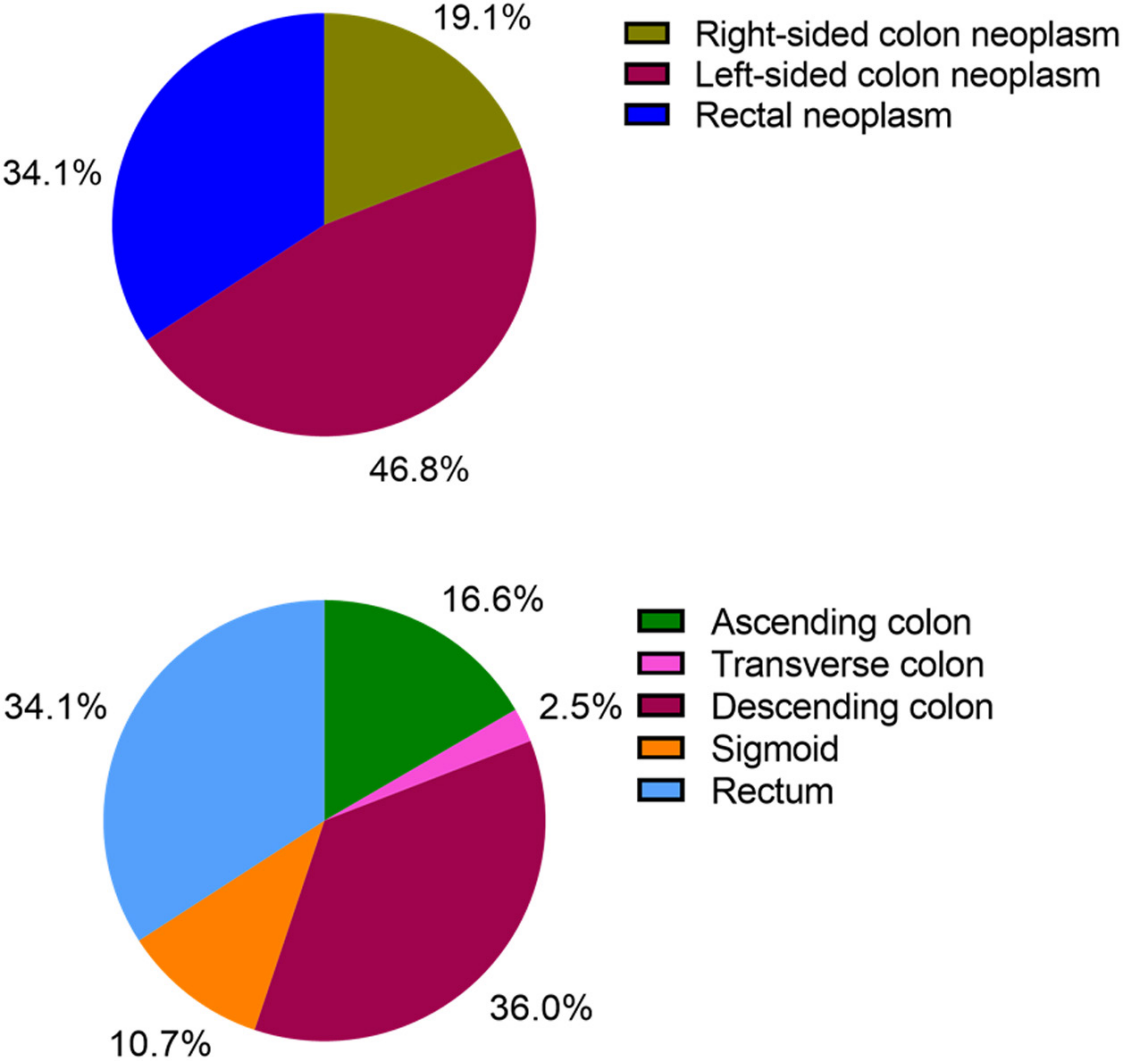
Anatomical location distribution of advanced colorectal neoplasms.

Among the advanced colorectal neoplasms, 1063 (34.1%) were located in the rectum, 1455 (46.8%) on the left side, and 595 (19.1%) on the right side, as depicted in Figure 1A. A more detailed examination of locations revealed that 517 patients (16.6%) had neoplasms in the ascending colon, while 78 (2.5%) had neoplasms in the transverse colon. Additionally, 1121 patients (36.0%) had neoplasms in the descending colon, 334 patients (10.7%) in the sigmoid colon, and 1063 patients (34.1%) in the rectum, as illustrated in Figure 1B.

### 3.3 Association between advanced colorectal neoplasm location and different patient factors

Table 2 summarizes the associations between various patient factors and the location of advanced colorectal neoplasms. The analysis of age groups reveals a statistically significant difference in the localization of advanced neoplasms among the four age groups (*p* < 0.001). In the age groups of 50–59, 60–69, and 70, the proportion of left‐side ANs was higher compared to right‐side and rectal ANs. However, in the 39–49 age group, the proportion of ANs on the left and right sides was equal. Furthermore, the highest incidence of left‐sided ANs occurred in the 50–59 age group (53.9%), while the highest incidence of right‐sided ANs was in the 39–49 age group (37.3%). Among rectal neoplasms, the highest incidence was observed in the 70‐year‐old age group (38.1%), and incidence also increased with age. The distribution of CRC localization in the left and right colon and rectum significantly differed between sexes (48.7% in males; 16.6% and 34.7% vs. 43.6% for females; 23.1% and 33.3%).

**TABLE 2:** The advanced neoplasm location was evaluated by univariate method.

Variables (*N*%)	Left‐sided colon AN (*N* = 1455)	Right‐sided colon AN (*N* = 595)	Rectal AN (*N* = 1063)	*p*
Age				
39–49	19 (1.3%)	19 (3.2%)	13 (1.2%)	<0.001
50–59	269 (18.5%)	95 (16.0%)	135 (12.7%)	
60–69	769 (52.9%)	283 (47.6%)	548 (51.6%)	
≥70	398 (27.4%)	198 (33.3%)	367 (34.5%)	
Sex				
Female	528 (36.3%)	279 (46.9%)	403 (37.9%)	<0.001
Male	927 (63.7%)	316 (53.1%)	660 (62.1%)	
Smoking status				
Never	987 (67.8%)	441 (74.1%)	717 (67.5%)	0.020
Former	82 (5.7%)	36 (6.1%)	67 (6.3%)	
Current	386 (26.5%)	118 (19.8%)	279 (26.2%)	
Drinking frequency				
Never	1016 (69.8%)	448 (75.3%)	736 (69.2%)	0.021
Ever	439 (30.2%)	147 (24.7%)	327 (30.8%)	
History of Appendicitis or appendectomy				
No	1380 (94.8%)	563 (94.6%)	1000 (94.1%)	0.698
Yes	75 (5.2%)	32 (5.4%)	63 (5.9%)	
FHCRC				
No	1335 (91.8%)	542 (91.1%)	1004 (94.4%)	0.013
Yes	120 (9.2%)	53 (8.9%)	59 (5.6%)	
History of Cholecystitis or cholecystectomy				
No	1374 (94.4%)	552 (92.8%)	993 (93.4%)	0.311
Yes	81 (5.6%)	43 (7.2%)	70 (6.6%)	
BMI				
<25	750 (51.5%)	334 (56.1%)	561 (52.8%)	0.168
≥5	705 (48.5%)	261 (43.9%)	502 (47.2%)	

Abbreviations: AN, advanced neoplasm; BMI, body mass index; FHCRC, Family history of CRC in FDR.

Additionally, smoking (*p* = 0.02) and alcohol consumption (*p* = 0.021), two well‐established risk factors for colorectal cancer, also showed statistical significance regarding AN location. The incidence of left‐sided ANs was higher in smokers than in never‐smokers (49.3% vs. 46.0%), while the incidence of right‐sided ANs was higher in non‐smokers than in smokers and ex‐smokers (20.6% vs. 19.5% vs. 15.1%). The lowest incidence of rectal ANs was found among individuals who had never smoked. Similarly, patients who consumed alcohol had a higher incidence of left‐sided ANs and rectal ANs (48.1% and 35.8%), while patients who never drank alcohol had a higher incidence of right‐sided ANs (20.4%).

A previous history or family history of colorectal cancer was associated with different distributions of AN localization (*p* = 0.013). It was observed that patients with a history of colorectal cancer in first‐degree relatives had a higher incidence of both left and right‐side ANs than those without such a history. However, the incidence of rectal ANs was lower in patients with a family history of colorectal cancer compared to those without.

There was no statistically significant difference between the history of cholecystitis and cholecystectomy (*p* = 0.311) or appendicitis and appendectomy and the localization of advanced colorectal neoplasms (*p* = 0.698). BMI showed a similar pattern (*p* = 0.168).

### 3.4 Further analysis of risk factors for different anatomic sites of ANs

**FIGURE 2:**
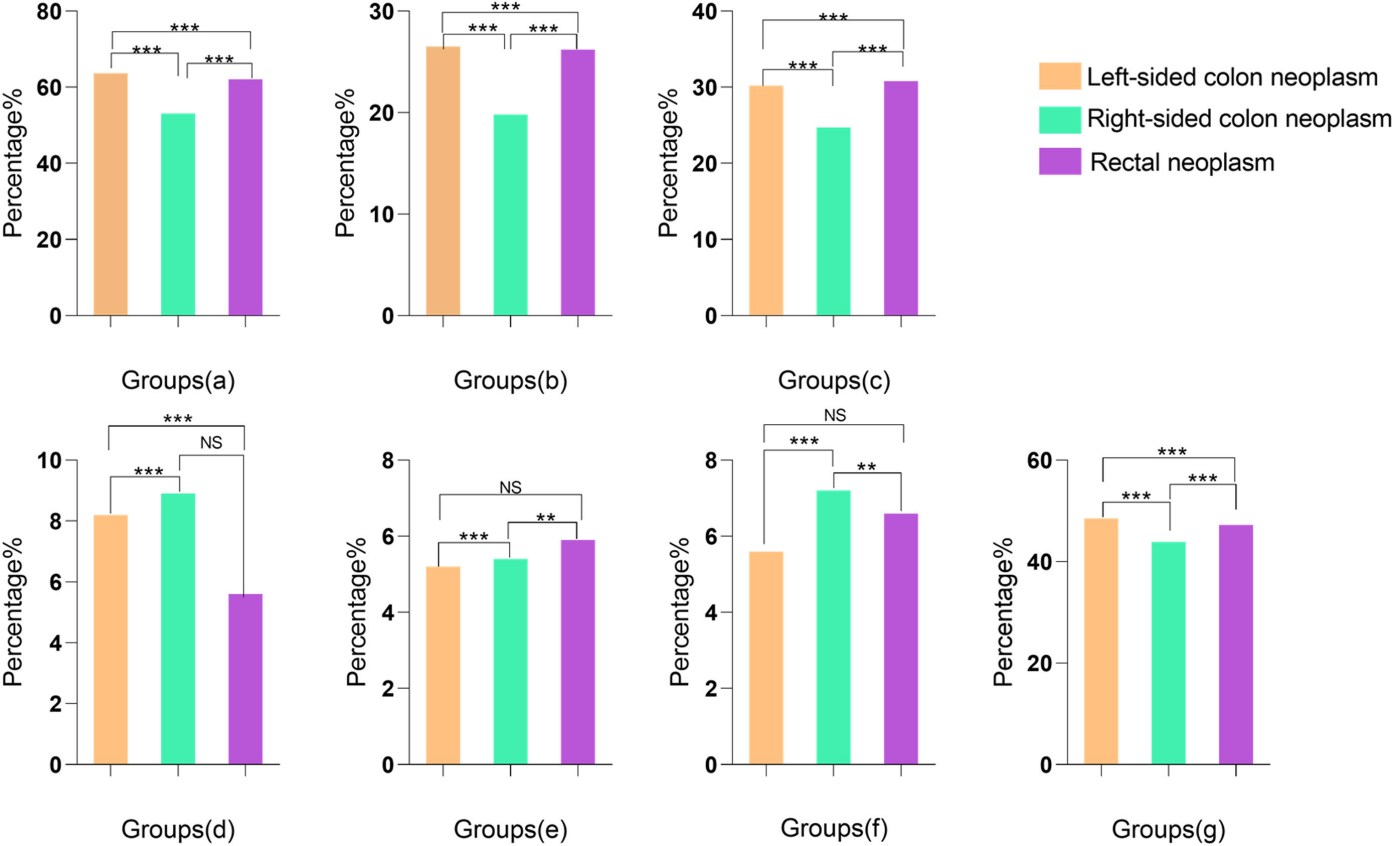
Comparison of population proportions and associated risk factors among all anatomical sites and among subgroups of anatomical sites. (A) Sex (male); (B) smoking (current); (c) drinking (Ever); (D) family history of CRC in FDR; (E) history of appendicitis or appendectomy; (F) history of cholecystitis or cholecystectomy; (G) BMI. **p<0.01; ***p<0.001; NS, no significance.

According to the initially included factors in our analysis, we aimed to better understand their potential impact on different AN locations. Consequently, we proceeded to examine the percentage of patients with identified risk factors within each subgroup based on anatomical site. We determined the corresponding *p*‐values to compare the risk factors among the three groups and within each pair of anatomical site subgroups. In the case of gender, our analysis specifically focused on male patients, and the results are presented in Figure 2A–G.

Furthermore, we further classified the anatomical sites of ANs and found that ANs in the descending colon accounted for the highest proportion, followed by rectal advanced neoplasms, while transverse colon advanced neoplasms accounted for the smallest proportion. The included risk factors remained statistically significant across different anatomical sites, as illustrated in Table 3.

**TABLE 3:** Distribution of advanced colorectal neoplasm in various locations.

Variables	*N* (%)					*p*
	Ascending colon (*N* = 517)	Transverse colon (*N* = 78)	Descending colon (*N* = 1121)	Sigmoid (*N* = 334)	Rectum (*N* = 1063)	
Age						
39–49	12 (2.3%)	7 (9.0%)	16 (1.4%)	3 (0.9%)	13 (1.2%)	<0.001
50–59	80 (15.5%)	15 (19.2%)	217 (19.4%)	52 (15.6%)	135 (12.7%)	
60–69	254 (49.1%)	29 (37.2%)	591 (52.7%)	178 (53.3%)	548 (51.6%)	
≥70	171 (33.1%)	27 (34.6%)	297 (26.5%)	101 (10.5%)	367 (34.5%)	
Sex						
Female	239 (46.2%)	40 (51.3%)	366 (32.6%)	162 (48.5%)	403 (37.9%)	<0.001
Male	278 (53.8%)	38 (48.7%)	755 (67.4%)	172 (51.5%)	660 (62.1%)	
Smoking status						
Never	379 (73.3%)	62 (79.5%)	730 (65.1%)	257 (76.9%)	717 (67.5%)	<0.001
Former	34 (6.6%)	2 (2.6%)	65 (5.8%)	17 (5.1%)	67 (6.3%)	
Current	104 (20.1%)	14 (17.9%)	326 (29.1%)	60 (18.0%)	279 (26.2%)	
Drinking frequency						
Never	384 (74.3%)	64 (82.1%)	763 (68.1%)	253 (75.7%)	736 (69.2%)	0.002
Ever	133 (25.7%)	14 (17.9%)	358 (31.9%)	81 (24.3%)	327 (30.8%)	
FHCRC						
No	469 (90.7%)	73 (93.6%)	1022 (91.2%)	313 (93.7%)	1004 (94.4%)	0.018
Yes	48 (9.3%)	5 (6.4%)	99 (8.8%)	21 (6.3%)	59 (5.6%)	

Abbreviation: FHCRC, Family history of CRC in FDR.

## 4 DISSUSION

The colorectal cancer rate in China is higher compared to global averages,^15^ and it has the highest number of cases.^16^ However, the specific epidemiological characteristics of colorectal cancer in the Chinese population, especially when stratified by anatomical sites, remain unclear. The right colon, left colon, and rectum originate from different embryonic origins,^17^, ^18^ resulting in varying pathogenic factors, clinical presentations, and prognostic factors associated with the development of colorectal cancer in these different anatomical regions. Therefore, conducting epidemiological studies on advanced colorectal neoplasms in a site‐specific manner holds greater significance. First and foremost, this study reveals that the incidence of advanced colorectal neoplasms on the left side is higher than that of rectal advanced colorectal neoplasms and significantly higher than that of the right colon. This finding aligns with previous reports from China and other Asian countries, which have consistently shown a higher prevalence of advanced colorectal neoplasms on the left side compared to the right side.^19^, ^20^, ^21^ However, when compared to results reported in Western countries, it becomes evident that the proportion of right‐side CRC is much greater in European and American countries than left‐side CRC.^22^ Additionally, this study reported a higher proportion of rectal advanced colorectal neoplasms compared to the 20%–30% reported in European and American countries.^23^

These differences have their reasons. Firstly, there are genetic differences among different ethnic groups,^21^ and some genes such as L1TD1, EFCAB2, PPP1R21, SLCO2A1, and HLA‐G have been found to have more specific significance in Asian colorectal cancer. Notably, the HLA‐G gene has been shown in studies to be closely associated with the occurrence and progression of rectal cancer in Asia.^22^, ^23^, ^24^ Secondly, the varying lifestyles between the East and the West have a relatively significant impact on the incidence of colorectal cancer.^25^, ^26^ In Asian diets, the proportion of high‐calorie intake is relatively uniform, which is positively correlated with the incidence of colon cancer and negatively correlated with physical exercise. However, these factors have less influence on rectal cancer.^24^, ^25^ Therefore, this may be one of the reasons for the higher incidence of rectal cancer in Asia. However, in recent years, as a result of the Westernization of diets and the adoption of sedentary lifestyles, the incidence of colorectal cancer in China has been steadily increasing.

Additionally, colorectal cancer in different anatomical sites often presents with different clinical symptoms. Rectal cancer usually exhibits early symptoms such as changes in bowel habits and bleeding, which are relatively easier to notice, whereas colon cancer, especially right‐sided colon cancer, often has less apparent early clinical manifestations.^26^ In recent years, many countries have initiated organized colorectal cancer screening programs.^27^ Western developed countries launched screening programs earlier and achieved good screening results, thereby increasing the chances of detecting right‐sided colon cancer at an early stage. However, China introduced its colorectal cancer screening program relatively late, and the high‐risk population for colorectal cancer in China has a lower participation rate and compliance with colonoscopy screening.^28^ This may also be a contributing factor to the higher proportion of rectal cancer diagnoses in Asia.

Our study results are consistent with the “rightward shift” trend in colorectal cancer reported in China,^29^, ^30^ which has increased from 10.9% in the 1980s to 15.2% in the 1990s. In this study, the rate was 19.1%. The prevalence of advanced colorectal neoplasms on the left side is comparable to what has been observed in previous studies conducted in China and other Asian nations,^31^ despite a decrease from 71.2% in the 1980s and 66.7% in the 1990s to 34.1% in this study. This change may still be related to changes in lifestyle and dietary habits, including increased intake of animal proteins and high‐sugar foods, as well as a widespread decrease in physical activity.^32^ Additionally, according to a Chinese epidemiological study on colorectal cancer from 1990 to 2009, the detection rate of right‐sided colon and rectal cancer increased more than that of left‐sided colon cancer.^33^ It can be reasonably predicted that in the future, especially among the younger generation, as Western lifestyles are adopted and physical activity decreases, this trend will become even more pronounced.

In our study, it is evident that the prevalence of ANs at various anatomical sites is higher in men, consistent with findings from previous reports.^14^ This observation may be attributed to male androgens.^34^ The activation of the androgen receptor has been found to regulate the secretion of BMP‐related signals in intestinal stromal cells, which is one of the possible mechanisms for the higher incidence of colorectal cancer in men.^35^

In addition, we observed that the incidence of left‐sided and right‐sided advanced neoplasms (ANs) in the 39–49 age group is similar. Previous studies have indicated that right‐sided colorectal cancer (CRC) tends to have a worse clinical prognosis compared to left‐sided CRC.^36^, ^37^ This finding may imply that younger patients with early‐onset CRC are more likely to develop right‐sided CRC, which aligns with the poorer prognosis associated with early‐onset colorectal cancer.^38^, ^39^ After further subdividing the anatomical site, we can find that the largest proportion of advanced colorectal neoplasm on the right side is the advanced ascending colon neoplasms. Therefore, it is recommended that individuals at high risk under the age of 50 should undergo full colonoscopy rather than relying solely on sigmoidoscopy to improve the detection rate of advanced colorectal tumors, thereby enhancing treatment and survival.^40^

Moreover, to further ascertain the factors impacting the development of advanced colorectal neoplasms at distinct anatomical locations, this study conducted an analysis of various risk factors, encompassing smoking, drinking, and family history of CRC. The objective was to compare the relationships between different anatomical sites and these risk factors.

It was observed that the occurrence of advanced colorectal neoplasms on the left‐side and rectal ANs was higher among smokers compared to non‐smokers. Among specific anatomical sites, transverse ANs had the highest prevalence, followed by the rectum, consistent with previous findings.^24^, ^41^ Smoking has been associated with specific genetic alterations, including MSI‐H (microsatellite instability‐high), CIMP (CpG island methylation phenotype), and mutated BRAF genes,^42^ which occur more frequently in the ascending colon compared to the rectum. These genetic changes might explain the varying relationships between smoking and the risk of colorectal cancer at different locations.^43^ However, in our study, we did not observe an association between smoking and right‐sided tumors; instead, it was associated with left‐sided tumors. This difference could possibly be attributed to several factors. First, it's worth noting that our study focused on a specific population within a particular geographic region. In contrast to existing research on the relationship between genes, smoking, and tumor location, our study had a smaller sample size. Additionally, the previous studies rigorously screened the individuals included, attempting to eliminate confounding factors like the influence of medications. In contrast, our study relied on questionnaire responses, which may have introduced other influences, such as the impact of medications. Furthermore, the complex interplay of various risk factors, genetic backgrounds, and environmental factors can lead to variations in colorectal cancer characteristics across different regions. This highlights the need for further exploration. But it is suggested that smoking cessation can reduce the occurrence of left ANs.^44^

Alcohol can be metabolized into biologically active chemicals, such as acetaldehyde. The accumulation of these compounds can suppress the body's cellular antioxidant defense system and induce DNA changes, contributing to the development of colorectal cancer (CRC).^45^, ^46^ Existing research indicates that ethanol and acetaldehyde may alter metabolic pathways and cellular structures, thereby increasing the risk of developing colon cancer.^47^ Alcohol is a well‐established risk factor for CRC according to existing research. However, previous studies have not shown a significant difference in anatomical location between colon and rectal CRC.^48^ In contrast, our findings indicate that advanced neoplasms in the colon are more prevalent than in the rectum, with alcohol displaying varying significance across different colon locations. The incidence of left‐colon advanced neoplasms was notably higher than that of right‐sided ones, and the descending colon had the highest proportion of left‐colon advanced neoplasms. This aligns with findings from a Japanese study.^49^ These insights may offer valuable guidance for colorectal cancer screening, particularly among individuals who consume alcohol.

Previous studies have indicated that both appendectomy and gallbladder removal are associated with an increased risk of colorectal cancer,^50^, ^51^, ^52^ particularly on the right side.^53^ The research suggests that postcholecystectomy changes in bile acid metabolism and disruptions in gut microbiota may be contributing factors to the development of colorectal cancer.^54^ Furthermore, results from a study conducted in China suggest that the gut microbiota may play a crucial role in the development of colorectal cancer induced by appendectomy.^55^ However, our data did not fully support this conclusion, possibly because some of our positive patients had a history of appendicitis or cholecystitis rather than surgical removal of these organs. Nevertheless, subgroup analyses of different anatomical sites among patients with a positive history revealed statistical significance in the context of appendectomy or gallbladder disease between left and right advanced colon neoplasms and between right‐sided and rectal advanced neoplasms. Therefore, further investigation is warranted to understand the significance of a history of gallbladder or appendix‐related issues.

Our findings suggest that individuals with close relatives previously diagnosed with colon cancer have a significantly higher probability of developing advanced colon neoplasms on both the left and right sides. Existing research supports the idea that individuals with a family history of colorectal cancer are at an increased risk of developing CRC. Other studies have also indicated a strong association between a family history of colorectal cancer and the risk of right‐sided colon cancer. A family history of CRC may be linked to various molecular subtypes, and specific types of colorectal cancer may comprise multiple molecular subtypes, such as LINE‐1 methylation and MSI‐low for cancers with high MSI. These molecular subtypes can vary along the gastrointestinal tract and may contribute to the anatomical variations observed in families with a history of colorectal cancer.

Previous studies have indicated that being overweight and obese are potential risk factors for colorectal cancer.^56^, ^57^, ^58^ However, our study results reveal that being overweight and obese are not significantly associated with the anatomical heterogeneity of advanced colorectal neoplasms. Nevertheless, subgroup comparisons of overweight and obese individuals across different anatomical sites show significant anatomical heterogeneity in left‐sided advanced colorectal neoplasms, right‐sided advanced colorectal neoplasms, and rectal advanced colorectal neoplasms. Existing research has shown a positive correlation between colon cancer and body mass index (BMI), but the incidence of rectal cancer is not significantly related.^25^ Furthermore, in recent years, some European countries have observed a decline in the proportion of colon cancer patients, which is attributed to the promotion of healthy habits and dietary improvements.^59^ Our findings also support this perspective. Many confounding factors may influence body weight, such as racial disparities and gender differences,^57^, ^60^ warranting further research.

Our study investigated the characteristics of different anatomical locations in late‐stage colorectal neoplasms and estimated the associations between the proportions of patients in each anatomical site subgroup and risk factors. This helped clarify the differences among late‐stage colorectal neoplasms in different positions, addressing the lack of relevant information in our country. However, our study has some limitations. Firstly, the number of participants included in the study was relatively small. Secondly, the collection of patient information relied mainly on questionnaire surveys, which may introduce recall bias and be susceptible to subjective influences in the data. Thirdly, due to the incompleteness of the questionnaire and screening content, some important information about patients, such as medication history, genetic testing, and clinical prognosis, could not be obtained, potentially impacting the analysis results.

## 5 CONCLUSION

In China, among advanced colorectal neoplasms, the largest proportion is found in the left‐sided colon, followed by rectal neoplasms, while right‐sided advanced colorectal neoplasms have the smallest proportion but show a continuous increasing trend. Among patients with advanced colorectal neoplasms, males hold a dominant position. Furthermore, age, alcohol consumption, smoking, and a family history of colon cancer in first‐degree relatives are significantly associated with the occurrence of advanced colorectal neoplasms.

Additionally, it has been observed that different anatomical subgroups of advanced colorectal neoplasms display varying gender distributions and medical histories. This suggests the need for tailored treatment approaches based on the distinct subtypes of advanced colorectal neoplasms in individual patients.

It is recommended to continuously improve screening programs and develop colorectal cancer screening and prevention strategies tailored to different anatomical sites to effectively reduce the incidence of colorectal cancer.

## AUTHOR CONTRIBUTIONS

**Kailong Zhao:** Conceptualization (lead); data curation (equal); resources (equal); supervision (equal). **Hongzhou Li:** Formal analysis (equal); software (equal). **Baofeng Zhang:** Data curation (equal). **Wenwen Pang:** Investigation (equal); visualization (equal). **Suying Yan:** Data curation (equal). **Xinzhu Zhao:** Data curation (equal). **Xinyu Liu:** Data curation (equal); validation (equal). **Wanting Wang:** Methodology (equal); resources (equal). **Qiurong Han:** Investigation (equal). **Yao Yao:** Formal analysis (equal). **Tianhao Chu:** Data curation (equal). **Zhiqiang Feng:** Conceptualization (equal). **Qinghuai Zhang:** Conceptualization (equal). **Chunze Zhang:** Formal analysis (equal); supervision (equal).

## FUNDING INFORMATION

This research was supported by the Key Research Project of Tianjin Science and Technology Support Program (Approval No. 19YFZCSY00420), Tianjin Natural Science Foundation (21JCZDJC00060, 21JCYBJC00180, and 21JCYBJC00340), Tianjin Key Medical Discipline Construction Project (Approval No. TJYXZDXK‐044A) and Hospital Management Research Project of Tianjin Hospital Association (Approval No. 2019ZZ07).

## CONFLICT OF INTEREST STATEMENT

The authors have no conflicts of interest to declare.

## ETHICS STATEMENT

The study was approved by the Tianjin Union Medical Ethics Committee and carried out according to the Helsinki Declaration.

## PARTICIPATION CONSENT STATEMENT

Participants signed an informed consent form prior to the start of the study.

## Data Availability

Because of the limitations of the ethical validation of patient data and anonymity, the data set analyzed in this study is not available to the public but can be obtained from relevant authors upon reasonable request. If you are interested in acquiring data, please contact Professor Chunze Zhang.
